# Expression of transient receptor potential vanilloid channels TRPV5 and TRPV6 in retinal pigment epithelium

**Published:** 2010-04-14

**Authors:** Brian G. Kennedy, Asad J. Torabi, Rafal Kurzawa, Stephen F. Echtenkamp, Nancy J. Mangini

**Affiliations:** 1 Department of Cellular & Integrative Physiology, Indiana University School of Medicine-Northwest, Gary, IN; 2 Department of Anatomy and Cell Biology, Indiana University School of Medicine-Northwest, Gary, IN; 3 Indiana University Northwest Gary, IN

## Abstract

**Purpose:**

Hydration and ionic composition of the subretinal space (SRS) is modulated by the retinal pigment epithelium (RPE). In particular calcium concentration (Ca^2+^) in the SRS varies with light exposure, and although this change is regulated by RPE transport activity, the specific transport proteins involved have yet to be defined. Two members of the transient receptor potential vanilloid family, TRPV5 and TRPV6, are calcium selective ion channels and are known to be expressed in calcium-transporting epithelial tissues. The present work characterizes of TRPV5 and TRPV6 in RPE.

**Methods:**

Reverse transcriptase PCR was used to examine the presence of *TRPV5* and *TRPV6* mRNA in cultured human RPE. Protein expression was assessed by western blotting using TRPV5- and TRPV6-specific antibodies. Immunocytochemistry was employed to examine subcellular localization of TRPV5 and TRPV6 in frozen, formaldehyde-fixed sections of native RPE–choroid tissue and in cultured human RPE monolayers. Finally, TRPV5/TRPV6 activity was assessed in cultured RPE, using Ca^2+^ indicator dyes to follow [Ca^2+^]_i_ as a function of changes in [Ca^2+^]_o_ with and without addition of the TRPV5/TRPV6 inhibitor ruthenium red.

**Results:**

Direct sequencing of PCR DNAs documented the presence of *TRPV5* and *TRPV6* transcripts in human RPE. Immunocytochemistry showed that TRPV5 and TRPV6 are expressed in native RPE–choroid tissue with strong immunoreactivity for both channels on the apical as well as the basal plasma membranes. Immunostaining for both channels was also positive in monolayers of cultured RPE cells. In cultured cells subcellular localization was variable with immunoreactivity present in the cytoplasmic domain as well as on the plasma membrane. Plasma membrane staining was increased with phagocytosis. The reported molecular weight of the core protein for both TRPV5 and TRPV6 is about 75 kDa, with the expected size of the glycosylated proteins in the range of 85–100 kDa. Western blot analysis of TRPV6 in RPE detected a distinct band at approximately 85 kDa, with another strong band at approximately 60 kDa. A similar pattern was seen for TRPV5, with strong bands at 82 kDa and 71 kDa. In live-cell imaging experiments, [Ca^2+^]_i_ was lower in the presence of the TRPV5/TRPV6 inhibitor ruthenium red.

**Conclusions:**

RPE expresses the epithelial calcium channels TRPV5 and TRPV6, the most calcium-selective channels of the TRP superfamily. Present findings suggest that these channels could function in RPE to mediate calcium influx from SRS and thus regulate changes in SRS calcium composition that accompany light/dark transitions.

## Introduction

The retinal pigment epithelium (RPE) is a monolayer of cells situated between the sensory retina and its choroidal blood supply. The RPE supports retinal photoreceptors by performing typical epithelial functions, such as water transport, intracellular and extracellular regulation of ion activity, and transepithelial solute exchange. The RPE also performs ocular-specific functions, such as re-isomerization of 11-cis retinal, light absorption, and phagocytosis of photoreceptor outer segments [1,2].

Because the apical surface of the RPE is in direct contact with photoreceptor outer segments, it serves to delineate a restricted extracellular space, known as the subretinal space (SRS). Photoreceptor and RPE membrane transport activity controls volume and ionic composition in the SRS. In particular, light-dependent changes in the SRS calcium concentration are modulated by RPE transport activity [1,3-6]. The specific calcium transport proteins that regulate the SRS calcium content have yet to be identified.

Extensive work has described both channel-mediated [7-14] and carrier-mediated [6,15-20] calcium transport mechanisms in the RPE. This transport activity ultimately controls both extracellular (SRS) and intracellular RPE calcium concentrations. Intracellular calcium is known to regulate growth factor secretion [1,7,12,21], phagocytosis [1,7,13,22,23], ion exchange [1,7,14,24-27], and water transport [26,28] in the RPE. Expression of several different calcium-selective channels has been described in the RPE. Both L-type [9-14] and T-type [9] voltage-gated calcium channels have been characterized in the RPE. In addition, the presence of fairly nonselective calcium channels of the transient receptor potential canonical (TRPC) family has also been documented in the RPE. TRPC1 [29,30] and TRPC4 [30] are reportedly expressed in the human retinal pigment epithelial cell-19 (ARPE-19) cell line, while TRPC1, TRPC4, and TRPC7 have also been detected in adult human RPE [30].

The TRP superfamily is a family of cation-selective ion channels that primarily have been identified based on sequence homology. The members of this superfamily provide a wide range of functions in multiple cell types. All of these channels are cation selective, with the relative calcium selectivity varying widely. The most calcium-selective channels in the TRP superfamily are members of the transient receptor potential vanilloid subfamily (TRPV), TRPV5 and TRPV6, with a calcium–sodium selectivity of more than 100:1 [31]. TRPV5 and TRPV6 (also known as ECaC1/CaT-2 and ECaC2/CaT-1, respectively) are predominantly expressed in epithelial tissues [31].

The purpose of the present work was to determine if the calcium ion-selective channels TRPV5 and TRPV6 are expressed in human RPE (hRPE) and whether they function to mediate calcium transport in this tissue.

## Methods

### Cell culture

Cell cultures were established from adult human donor eyes obtained from the National Disease Research Interchange (Philadelphia, PA). Institutional Review Board (Indiana University, Purdue University, Indianapolis, IN) approval for the use of human donor tissue was obtained. No ocular disease was reported for any donor. As described previously [15,16,32-38] to initiate cultures, small patches of RPE–choroid tissue (about 0.5 cm^2^) were dissected and placed RPE-side down in 60-mm culture dishes (Falcon Primaria; BD Biosciences, Bedford, MA). RPE cells, which grow out from the explant, were harvested by trypsinization and then maintained in 25-cm^2^ flasks (Falcon Primaria). Cells were cultured in Eagle's Minimum Essential Medium with Earle's salts (Mediatech Cellgro; Fisher Scientific, Pittsburgh, PA) supplemented with 15 mM sodium bicarbonate (44 mM), 10% fetal calf serum (Aleken Biologicals, Nash, TX), 5% newborn calf serum, essential and nonessential amino acids, 4 mM l-glutamine, 0.5 μg/ml amphotericin B, and 10 μg/ml gentamicin at 37 °C with 5% CO_2_ in air. Except as noted, chemicals for cell culture were obtained from Sigma Life Science, St. Louis, MO. Cells were subcultured at 1–2-week intervals by trypsinization. These cells are reactive for cytokeratin-18 [16] and can attain a hexagonal packing array with pigmentation [33]. These cells have been used to examine the Na^+^:Ca^2+^ exchange protein [16,17,33], plasma membrane calcium ATPase [15], arrestin [33], transthyretin [38], volume regulation [32], Na-K-Cl co-transport [35], creatine kinase [36], and p-glycoprotein [37] in RPE. In the present study, cell cultures from five different donors were used (donor R, 72-year-old Caucasian male, passage numbers 83 to 184; donor U, 82-year-old Caucasian male, passage numbers 53 to 154; donor W, 47-year-old Caucasian female, passage numbers 27 to 80; donor G, 10-year-old Caucasian female, passage 3; and donor F, 68-year-old Caucasian male, passage 7). Our results examining TRPV5 and TRPV6 expression by western blotting, immunocytochemistry and live-cell Ca^2+^-imaging were consistent across cultures.

### Reverse transcriptase PCR

Analysis was performed on cDNA prepared from cultured hRPE (donor cultures G and F) and from freshly isolated RPE (70-year-old Caucasian female). Primer sequences were from Hoenderop et al. [39] and were designed to span an intron at the genomic level. For *TRPV5* (ECaC1, CAT-2) forward 5′-GGG GAC CGC TGG TTC CTG CGG-3′ and reverse 5′-TCA AAA ATG GTA GAC CTC CTC TCC-3′ primers, the expected size of amplified DNA product was 312 bp. For *TRPV6* (ECaC2, CAT-1) forward 5′-GGG GAC CGC TGG TTC CTG CGG-3′ and reverse 5′-AGA TCT GAT ATT CCC AGC TCT-3′ primers, the expected size of amplified DNA product was 301 bp. PCR reactions were performed in a volume of 25 μl using High Fidelity PCR Master (Roche Applied Science, Indianapolis, IN), a GeneAmp PCR System 2700 (Applied Biosciences, Foster City, CA), and using the following cycling conditions: initial denaturation at 94 °C for 2 min, then 35 cycles of 94 °C for 10 s, 55 °C for 30 s, 72 °C for 1 min, followed by 72 °C for 7 min. PCR products were analyzed by electrophoresis on 2% agarose-1x Tris-acetate-EDTA (TAE) gels (8 min at 210 V), using a Rapid Agarose Gel Electrophoresis System (RGX60; Biokey American Instrument, Inc., Portland, OR). PCR DNAs were purified (Hi-Pure Purification Kit; Roche Applied Science), and the identity as TRPV5/TRPV6 transcripts was confirmed by direct sequencing (Cancer Research Center DNA Sequencing Facility, University of Chicago, Chicago, IL).

### Western blot analysis

Western blot analysis was performed on whole cell lysates that were obtained from confluent monolayers growing in 25-cm^2^ culture flasks. Monolayers were washed free of culture medium with standard buffer (140 mM NaCl, 5 mM KCl, 1 mM MgCl_2_, 2 mM CaCl_2_, 5 mM glucose, 15 mM HEPES, 7 mM Tris base, at pH 7.4 and 290 mOsmol/l) and then harvested by scraping into standard buffer. Cells were collected by centrifugation at 1,500× g for 8 min. The pellet was suspended in standard buffer, sampled for protein determination, and then dissolved in electrophoresis buffer (2% sodium dodecyl sulfate, 10% glycerol, 200 mM HEPES at pH 6.8, 1 mM EDTA [EDTA], 0.1% bromphenol blue, and protease inhibitor cocktail [Complete Roche Diagnostics, Indianapolis, IN, and Protease Inhibitor Cocktail; Sigma, St. Louis, MO). Samples in electrophoresis buffer were stored at –20 °C until used. For electrophoresis, samples were heated for 10 min at 37 °C and then resolved on 6% Laemmli gels [40]. Protein was transferred to polyvinylidene fluoride membranes (Immobilon P; Millipore, Billerica, MA) by semi-dry blotting. For immunodetection, membranes were first blocked for 1.5 h at room temperature with Super Block (Thermo Scientific, Pierce Research Products, Rockford, IL). Incubation with the primary antibody was for 1.5 h at room temperature. Primary antibodies were diluted in high salt Tris-buffered saline (500 mM NaCl, 25 mM Tris base, pH 7.5) containing 0.4% blotting grade nonfat dry milk (Bio-Rad, Hercules, CA) and 0.2% Tween-20 (Sigma). Secondary antibodies (Vector Labs, Burlingame, CA) were alkaline phosphatase conjugated and were diluted (1:5,000) in the same solution used for the primary antibodies. Blots were reacted with secondary antibodies for 1.5 h at room temperature. Reactive bands were visualized by chemiluminescence using CDP Star substrate (Tropix, Bedford, MA). The chemiluminescent signal was digitally captured with a Kodak Image Station 440cf (Carestream Health, Inc., Rochester, NY). Bands were analyzed and molecular weights estimated with 1D Image Analysis software (Carestream Health).

### Immunohistochemistry

Immunocytochemistry was performed on native human RPE–choroid patches as well as on cultured RPE monolayers. Native RPE–choroid tissue was obtained from postmortem human eyes and fixed by immersion in 4% paraformaldehyde in PBS (time into fix approximately 24 h postmortem). PBS composition was 130 mM NaCl, 2.5 mM KCl, 8 mM Na_2_HPO_4_, and 1.5 mM KH_2_PO_4_. RPE–choroid patches were dissected from the eyes, placed in Optimal Cutting Temperature (OCT; Sakura Finetek, Torrance, CA) media, and 10 µm-thick sections were obtained by cryostat. Before antibody incubation, sections were permeabilized with 0.1% Triton X-100 in PBS and then treated with 0.25% KMnO_4_ in PBS for 15 min at room temperature to minimize tissue autofluorescence [36]. Sections were next blocked with 10% goat serum (2 h at 37 °C), reacted with primary antibody (overnight at 4 °C), blocked again as described above, and finally reacted with Alexa 488-labeled secondary antibodies (Invitrogen, Carlsbad, CA). Sections were mounted with Vectashield Mounting Medium (Vector Laboratories) containing TO-PRO-3 (Invitrogen) to stain nuclei and were imaged by confocal microscopy (Olympus FV 300; Olympus, Tokyo, Japan).

Immunohistochemistry of cultured hRPE was performed in monolayer cultures grown on Permanox chamber slides (Lab-Tek; Nunc, Fisher Scientific). Culture medium was washed off with standard buffer; cells were fixed for 30 min at room temperature with 4% formaldehyde in PBS and then treated with permeabilization buffer (100 mM Hepes, 10 mM EGTA, 5 mM MgCl_2_, 0.1% Triton X-100, pH 7.0). Blocking solution (10% donkey or goat serum [Jackson ImmunoResearch Laboratories, West Grove, PA] in PBS) was applied before incubations with primary and secondary antibodies. Primary antibodies were diluted in PBS and incubated at 4 °C overnight. Secondary antibodies, also diluted in PBS, were conjugated to Alexa dyes (Invitrogen). Secondary antibody incubations were for 2 h at 37 °C. Nuclei were visualized with TO-PRO-3 (Invitrogen; 10-min incubation at a final concentration of 1 µM). Similar to native tissue cytochemistry, cell cultures were imaged by confocal microscopy (Olympus FV 300; Olympus).

For zymosan experiments, cells were washed free of culture medium and pre-incubated in standard buffer for 30 min at 37 °C. Standard medium containing zymosan (Sigma) was then added for 10 min at 37 °C. The reaction was stopped with 4% formaldehyde in PBS fixation, and immunohistochemistry was continued as described above.

### Intracellular calcium imaging

TRPV5/TRPV6 activity was assessed in RPE monolayers by measuring changes in intracellular calcium ([Ca^2+^]_i_) with and without the addition of ruthenium red. Relative changes in [Ca^2+^]_i_ were monitored by single-cell digital fluorescence imaging in cells loaded with fluo-3. Preparation of fluo-3 and the method of dye-loading were performed as described previously [16]. Briefly, hRPE cells were plated at low density on four-well chamber cover glass slides (Lab-TEK; Nunc) and were studied 2–4 days after plating. Cells were dye loaded with 8.9 μM fluo-3 AM (Invitrogen, Molecular Probes, Eugene OR) in control extracellular solution (120 mM NaCl, 2 mM KCl, 0.5 mM CaCl_2_, 1.0 mM MgCl_2_, 5 mM glucose, 23 mM NaHCO_3_, and 15 mM HEPES, adjusted to pH 7.4 with NaOH) in the presence of 0.08% Pluronic F-127 detergent (cat no. P3000MP, Invitrogen) and 0.8% dimethyl sulfoxide (DMSO, Sigma). Loading was in the dark at room temperature (23 °C) for a minimum of 30 min followed by rinsing in control extracellular solution. During recordings extracellular Ca^2+^ was varied (0.5 mM, 1 mM, 2 mM, and 5 mM) with and without the addition of ruthenium red (final concentration 100 μM), using a 16-channel high-performance perfusion system with automated flow control (flow rate approximately 3 ml min^−1^; Bioscience Tools, San Diego, CA). Cells were imaged with an Olympus IX71 inverted microscope equipped with a 75 W xenon burner, a blue excitation fluorescence mirror unit (U-MWB2; Olympus America Inc., Center Valley, PA), and a Retiga EXi camera (QImaging, Surrey, Canada). Hardware control, data acquisition, and analysis were through IPLAB v. 4.04 scientific software (IPLAB Motion Control and Ratio-Plus, Scanalytics; Becton Dickinson and Co., Exton, PA). An Olympus UApo/340 20X/0.17 objective was used for all studies. In a typical experiment responses were collected from five to 15 cells per field of view. The software was used to circumscribe regions of interest that overlaid single RPE cells. Calcium responses were determined by spatially averaging the fluo-3 fluorescence in each cell. Studies were performed using primary cultures of adult hRPE from three different donors (donor R, passage 152; donor U, passage 122; donor W, passage 61). Our data examining the effect of ruthenium red on intracellular calcium were consistent across cultures. Numerical data from Ca^2+^-imaging experiments are presented as mean±standard error of the mean of averaged measurements from several cells in a representative experiment. Statistical significance (p<0.01) was assessed using two-way ANOVA with repeated measures (KaleidaGraph v.4.03; Synergy Software, Reading, PA).

### Antibodies

All antibodies used in the present study are commercially available. Anti-TRPV5 antibodies were KL362 (Biomol International, Enzo Life Sciences, Plymouth Meeting, PA), CAT21A (Alpha Diagnostic International, Inc., San Antonio, TX), and 8515 (Everest Biotech, Upper Heyford, Oxfordshire, UK); Anti-TRPV6 antibodies were KL364 (Biomol International), CAT11A (Alpha Diagnostic International, Inc.), N16, and E16 (Santa Cruz Biotechnology, Santa Cruz, CA). KL362, KL364, CAT11A, 8515, N16, and E16 were used in immunocytochemical experiments, while 8515, 11A, KL364, and N16 were used in western blotting experiments.

Details on the antibodies used in the present work are from the respective manufacturers’ product data sheets. Briefly, KL362 is an affinity-purified rabbit polyclonal antibody raised against a peptide corresponding to amino acid residues 715–729 of human TRPV5 (GenBank Q8NDW5). Antibody CAT21A is an affinity-purified rabbit polyclonal antibody raised against a 20-amino acid peptide (ADI CAT21-P) located at the cytoplasmic C-terminus of rat TRPV5 and corresponding to amino acid residues 704–723 (GenBank NP_446239.1). Antibody 8515 is an affinity-purified goat polyclonal antibody raised against a peptide with sequence C-SHRGWEILRQNT (amino acid residues 697–708, GenBank NP_062815.2) from the internal region of the human TRPV5 protein. KL364 is an affinity-purified rabbit polyclonal antibody raised against a peptide corresponding to amino acid residues 715–725 of human TRPV6 (GenBank Q9H1D0). Antibody CAT11-A is an affinity-purified rabbit polyclonal antibody raised against an 18-amino acid peptide (ADI CAT11-P) located within the cytoplasmic N-terminus of rat TRPV6 and corresponding to amino acid residues 1–18 (GenBank NP_446138.1). N-16 is an affinity-purified goat polyclonal antibody raised against a peptide mapping near the N-terminus of human TRPV6. E-16 is an affinity-purified goat polyclonal antibody raised against a peptide mapping within an internal region of human TRPV6.

## Results

### Reverse transcriptase PCR analysis

**Figure 1:**
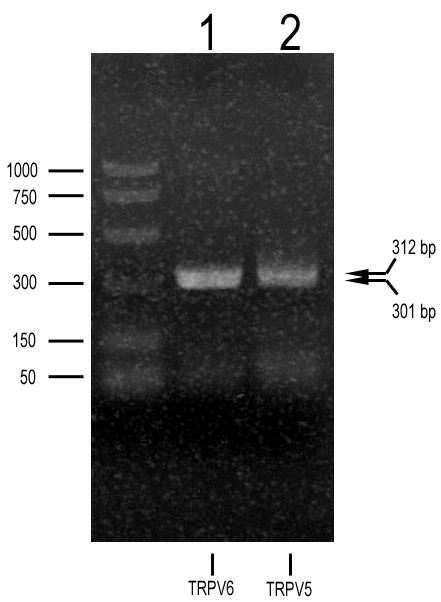
Reverse transcriptase PCR analysis of transient receptor potential vanilloid member 5 (*TRPV5)* and member 6 (*TRPV6)* expression in cultured human retinal pigment epithelium. Agarose gel electrophoresis of PCR products amplified with *TRPV5*- (lane 2) and *TRPV6*- (lane 1) specific primers. CDNA (cDNA) was from primary retinal pigment epithelium cultures established from donor tissue from a 10-year-old Caucasian female. Arrows to the right identify the expected size of the PCR DNA for *TRPV5* (312 bp) and for *TRPV6* (301 bp). Direct sequencing of the purified PCR DNAs confirmed the identities of the bands as *TRPV5* and *TRPV6* transcripts.

Representative results are presented in Figure 1. Lane 1 shows the product (301 bp) from amplification with *TRPV6*-specific primers, while lane 2 presents the product (312 bp) after amplification with *TRPV5*-specific primers. Both products are the size expected based on the respective primer sets. Direct sequencing confirmed the identities of the respective bands as *TRPV5* and *TRPV6*.

### Western blot analysis

**Figure 2:**
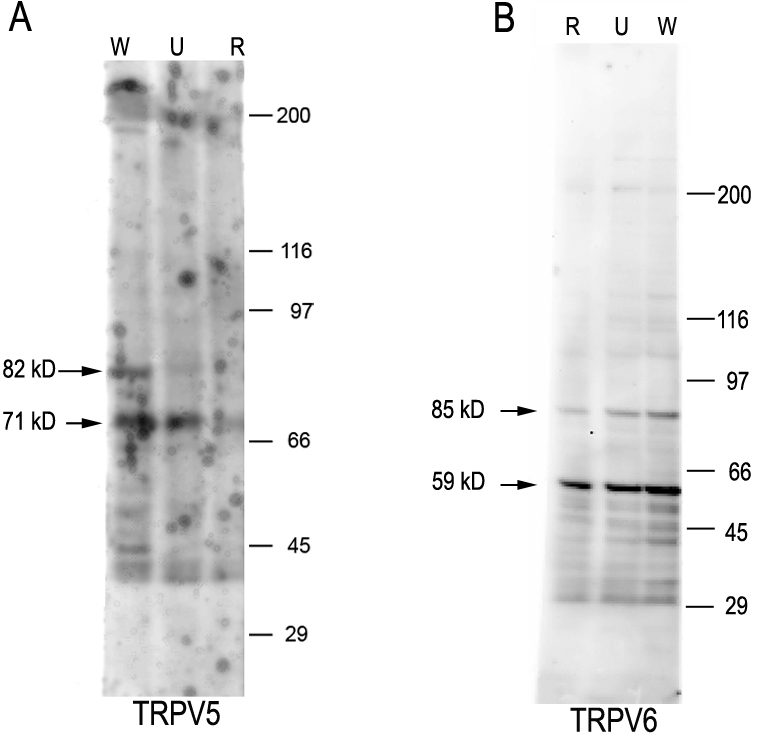
Western blot analysis of transient receptor potential vanilloid channel member 5 (TRPV5) and member 6 (TRPV6) protein expression in cultured human retinal pigment epithelium. **A**: Reactivity with anti-TRPV5 polyclonal antibody 8515 (final concentration 1 µg/ml). The secondary antibody was antigoat immunoglobulin G (IgG) conjugated to alkaline phosphatase (final dilution 1:5,000). Samples were from three different human primary cultures: R established from a 72-year-old Caucasian male and used at passage 72; U established from an 82-year-old Caucasian male and used at passage 42; W established from a 47-year-old Caucasian female and used at passage 16. **B**: Reactivity with an anti-TRPV6 affinity-purified polyclonal antibody 11A (final concentration 0.5 µg/ml). The secondary antibody was antirabbit IgG conjugated to alkaline phosphatase (final dilution 1:5,000). In **B**, culture R was used at passage 83, culture U at passage 53, and culture W at passage 27.

Western blot analysis was performed to determine if TRPV5 and TRPV6 protein are expressed at detectable levels by hRPE. Examples of western blots examining expression of TRPV5 and TRPV6 are presented in Figure 2. Whole cell lysates from three primary human donor RPE cultures were tested for both channels. For TRPV5 (Figure 2A) a prominent band was detected at about 70 kDa in all lysates examined. An additional higher band at approximately 82 kDa was most prominent in lane W and also weakly detected in lane U; this higher band was not detectable in the sample from donor R. For TRPV6 (Figure 2B) a strongly labeled band at approximately 60 kDa as well as a weaker band at approximately 85 kDa were detected in samples from all donors. The molecular weight of the core TRPV5 and TRPV6 proteins is approximately 75 kDa. In vivo, both proteins are glycosylated, with apparent molecular weights on sodium dodecyl sulfate–PAGE in the range of 80–100 kDa.

### Immunohistochemistry

**Figure 3:**
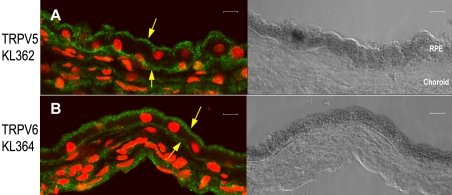
Immunolocalization of transient receptor potential vanilloid channels, TRPV5 and TRPV6, in frozen sections of native human retinal pigment epithelium/choroid tissue. Tissue orientation in all panels shows the retinal pigment epithelium (RPE) monolayer above the choroid tissue. In the fluorescent images (left two panels), TRPV5 immunoreactivity (**A**) and TRPV6 immunoreactivity (**B**) appear green; nuclei (red) were visualized with TO-PRO-3; corresponding differential interference contrast (DIC) images (panels on the right) show the pigmented RPE monolayers. In **A**, the anti-TRPV5 antibody used was KL362 (affinity-purified polyclonal antibody, final concentration 2.5 µg/ml). In **B,** the anti-TRPV6 antibody used was KL364 (affinity-purified polyclonal antibody, final concentration 2.5 µg/ml). The secondary antibody used in both panels was antirabbit immunoglobulin G (IgG) conjugated to Alexa 488 (final dilution 1:500). Down arrows in both panels identify prominent staining on or just below the apical plasma membrane; up arrows identify prominent staining along the basal plasma membrane. Sections are from a 47-year-old Caucasian female donor. The pattern of staining in these panels is representative of staining observed on sections from four different donors (three female, one male, ranging in age from 47 to 79 years). Scale bars in all panels represent 10 µm.

TRPV5- and TRPV6-specific antibodies were used to further examine whether TRPV5 and TRPV6 channels are expressed in human RPE and specifically to obtain information about their subcellular localization. Immunostaining was performed on frozen sections of human RPE–choroid tissue from four different adult donors, ranging in age from 47 to 79 years, and using two different anti-TRPV5 and anti-TRPV6 antibodies. Figure 3A shows reactivity for anti-TRPV5 in native human RPE–choroid tissue. Prominent staining is evident on or just below both apical and basal plasma membranes. Figure 3B shows a similar pattern for anti-TRPV6 immunoreactivity. This pattern of staining was observed on tissue sections from all four donors examined. The pattern of staining was consistent for the anti-TRPV5 antibodies KL362 and 8515 as well as for the anti-TRPV6 antibodies KL364, N-16, and 11-A.

**Figure 4:**
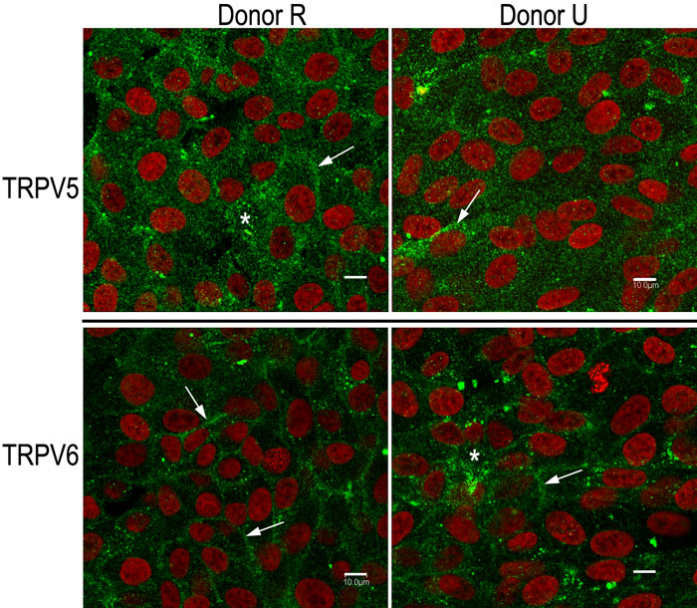
Immunoreactivity against transient receptor potential vanilloid channel member 5 (TRPV5) and member 6 (TRPV6), respectively is present on the plasma membrane as well as in the cytoplasm of cultured adult human retinal pigment epithelium. Donor cultures (R on the left and U on the right) are the same as those used for western blotting (Figure 2). Cells were plated (passage 146 for R and passage 116 for U) onto uncoated four-well glass chamber slides and grown for 3 days under standard culture conditions (described in the Methods). Monolayers were stained for TRPV5 (upper panels) using an affinity-purified, polyclonal, anti-TRPV5 antibody (8515, final concentration 5 µg/ml). Monolayers were stained for TRPV6 (lower panels) using an affinity-purified, polyclonal, anti-TRPV6 antibody (E-16, final concentration 2 µg/ml). The same secondary antibody was used with both primary antibodies: antigoat Alexa 488 (final dilution 1:500). Nuclei (red) were visualized with TO-PRO-3 (final concentration 2 µM). Scale bars in all panels represent 10 µm. Both plasma membrane (arrows) and prominent, punctuate, cytoplasmic (asterisk) staining is seen for both antibodies.

TRPV5 and TRPV6 immunoreactivity was also examined in cultured monolayers of hRPE. Staining was examined for both TRPV5 and TRPV6 in cultures established from three different donors. A typical example of staining observed in cultures from two donors is shown in Figure 4, and reactivity for both TRPV5 and TRPV6 is evident. Although reactivity localized to the plasma membrane is evident, significant staining is also present in the cytoplasmic compartment. In multiple independent experiments, this overall pattern held constant, although the relative extent of cytoplasmic versus membrane stain varied. A lack of clear membrane staining for TRPV5 channels due to a predominant cytoplasmic localization has been described previously [41,42]. Thus, it has been shown that a significant population of TRPV channels is held dormant in cytoplasmic vesicles in many cell systems, with channel activation dependent upon transient vesicle insertion into the plasma membrane [31,41-45].

### Regulation

**Figure 5:**
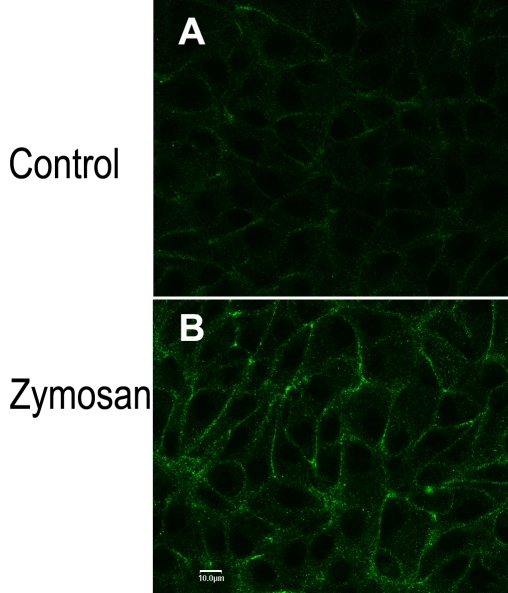
Transient receptor potential vanilloid channel, member 6 (TRPV6) localization to the plasma membrane increased after treatment with zymosan, a reagent that is phagocytized by the retinal pigment epithelium. Donor R cells were plated (passage 151) onto Permanox four-well chamber slides and grown for 7 days under standard culture conditions. Monolayers were rinsed free of media with standard buffer and equilibrated in that buffer for 30 min at 37 °C. Monolayers were exposed to either standard buffer (Control, upper panel) or standard buffer plus 2 mg/ml zymosan (Zymosan, lower panel) for a further 10 min at 37 °C. Reaction was stopped by formaldehyde fixation, and immunocytochemistry was performed. Primary and secondary antibodies were those used in Figure 4, bottom panel (TRPV6). Nuclei were stained with TO-PRO-3. The scale bar in panel **B** represents 10 μm and applies to both panels.

Salceda [22] characterized phagocytosis in frog RPE and showed that an increase in calcium uptake by the RPE was associated with phagocytosis of the reagent zymosan. This calcium uptake was inhibited by ruthenium red, a relatively nonselective inhibitor of calcium uptake that has been shown to block TRPV5 and TRPV6 channel activity [31,39,41,42,44,46]. Based on these earlier observations, we hypothesized that the zymosan-associated calcium influx could be mediated by activation of TRPV channels. Furthermore, work in other cell systems has shown that TRPV5 and TRPV6 channels can be activated by translocation from intracellular vesicles to the plasma membrane [31,41-45]. Figure 5A shows TRPV6 immunoreactivity in control hRPE monolayers. In parallel experiments, cells were exposed to zymosan for 10 min at 37 °C, then reacted with anti-TRPV6 (Figure 5B). Zymosan treatment was associated with a marked increase in plasma membrane-localized TRPV6 reactivity. This is consistent with a model postulating activation of TRPV6 in hRPE by movement of channels from intracellular vesicles to the plasma membrane.

### Activity

**Figure 6:**
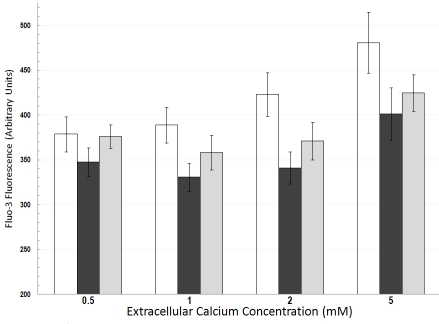
Composite data showing fluorescence measurements from fluo-3-loaded cultured human retinal pigment epithelium. Cells were examined before (open bars), during (black filled bars), and after recovery from (filled gray bars) exposure to 100 μM ruthenium red. The effect of ruthenium red was examined in the presence of different extracellular calcium concentrations (0.5, 1, 2, and 5 mM). Values are means±standard error of the mean from five cells (U donor, passage 152). Two-way ANOVA with repeated measures indicated that the lower calcium level in the presence of 100 μM ruthenium red was significant (p<0.01).

We hypothesized that TRPV activity plays a role in RPE calcium homeostasis. Cultured adult hRPE was loaded with fluo-3, and relative changes in intracellular free calcium were followed as a function of time with and without the addition of 100 μM ruthenium red, an inhibitor of TRPV channels. Experiments examined responses in cells from three different donors. Composite data from a representative experiment in Figure 6 show that intracellular calcium levels were lowered in the presence of the TRPV5/TRPV6 inhibitor; the effect was observed in the presence of different extracellular calcium concentrations (0.5, 1, 2, and 5 mM). These observations are consistent with involvement of TRPV5/6 in intracellular calcium homeostasis in RPE. Notably, previous work with keratinocytes [47] has also shown a TRPV6-mediated increase in intracellular calcium following elevation of extracellular calcium.

## Discussion

TRPV5 and TRPV6 are two homologous members of the TRP superfamily [31]. These two channels are predominantly expressed in epithelial tissues and presumably function in transcellular calcium transport [31]. Although TRPV5 is the major isoform expressed in kidney and TRPV6 predominates in intestine, the tissues co-express both isoforms. TRPV5 and TRPV6 have distinct physiologic properties that distinguish them from other members of the TRP superfamily. First, both channels are constitutively active at physiologic levels of membrane potential and intracellular calcium [31]. Research suggests channel activity is normally regulated by an exocytic mechanism that inserts channel-containing vesicles into the plasma membrane [41-45,48]. Second, TRPV5 and TRPV6 are the most calcium-selective channels of the TRP family, exhibiting a Ca:Na permeability ratio >100 [31].

Calcium as second messenger controls multiple critical functions in the RPE [7,12,13,21-28]. Plasma membrane transport proteins known to regulate intracellular calcium, including channels [7-14], ATPases [15], and exchangers [16,17], have been characterized in the RPE. Although the RPE catalyzes net transepithelial calcium transport [6], neither TRPV5 nor TRPV6 expression has been described in the RPE. This is notable given the fact that TRPV5 and TRPV6 are widely expressed in calcium-transporting epithelia [31,39,41,42,44-46,48]. The present work documents expression, activity, and regulation of TRPV5 and TRPV6 calcium channels in human RPE.

Reverse transcriptase PCR, western blot analysis, and immunocytochemistry all indicate that both TRPV5 and TRPV6 calcium channels are expressed in human RPE. Message for both *TRPV5* and *TRPV6* was detected in primary cultured RPE established from adult human donor tissue. Western blot analysis indicated that in cultured hRPE both channels were detected at the protein level. Immunohistochemistry was performed on both native and cultured human RPE. By immunohistochemical analysis TRPV5 and TRPV6 were expressed in both native and cultured hRPE. Additionally, at the subcellular level in native RPE–choroid preparations of adult hRPE, TRPV5 and TRPV6 channels were present on both apical and basal plasma membranes. Additionally, in cultured and native RPE, significant immunoreactivity for both channels were detected in the cytoplasmic domain. This is consistent with previous reports that hypothesize that the majority of TRP channels may be stored in cytoplasmic vesicles, with channel activation dependent upon vesicle insertion into the plasma membrane [31,41-45].

Salceda [22] documented that increased calcium uptake is associated with zymosan phagocytosis in frog RPE. This calcium uptake was inhibited by ruthenium red, a widely used, although not completely selective, inhibitor of TRPV5 and TRPV6 channels [31,39,41,42,44,46]. We hypothesized that at least part of the zymosan-associated calcium influx described by Salceda in that earlier study is mediated by TRPV channels. Thus, exposure to zymosan should activate TRPV channels in the RPE. Furthermore, because activation of TRPV channels is by insertion into the plasma membrane [31,41-45], we reasoned that exposure to zymosan should increase TRPV immunoreactivity in the RPE plasma membrane. This prediction was confirmed in cultures of adult hRPE whereby increased membrane-associated immunoreactivity of TRPV6 was observed after zymosan exposure (Figure 5).

Intracellular calcium was monitored with the calcium indicator dye fluo-3 to test for possible TRPV channel activity in cultured hRPE. TRPV6 activity has been shown to mediate an increase in intracellular calcium, secondary to increasing extracellular calcium [47]. Data in Figure 6 are consistent with these observations and show that the intracellular calcium concentration in cultured hRPE was sensitive to the TRPV calcium channel blocker ruthenium red.

Both L- and T-type calcium channels have been described in the RPE [8-14]. These calcium channels exhibit low calcium permeability at normal levels of membrane potential; channel permeability can be increased by membrane depolarization or by second messenger-induced changes in the channel activation threshold [7]. TRPV5 and TRPV6 channels are constitutively active at normal levels of membrane potential [31,47]. A model for TRPV5/TRPV6 activation proposes that the TRPV5/TRPV6-mediated increase in calcium permeability is dependent upon channel insertion into the plasma membrane [41,42,44,45]. In this regard it has been shown that phagocytosis in the RPE is associated with a ruthenium red-sensitive increase in calcium uptake [22]. The present work implicates TRPV6 in this calcium uptake and further indicates that TRPV6 activation, in RPE, could be mediated by channel insertion into the plasma membrane.

Calcium transport in the RPE could serve three distinct functions: regulation of intracellular calcium concentration, transepithelial calcium transport, and regulation of calcium concentration in the SRS. Direct evidence from fluo-3 measurements (Figure 6) as well as indirect immunocytochemical observations (Figure 5) indicates that TRPV5/TRPV6 channels can modulate calcium levels in the human RPE. TRPV5 and TRPV6 channels could function with L-type calcium channels to mediate calcium influx in RPE, thus contributing to intracellular calcium regulation in these cells. The relative importance of each channel type, either in the steady-state or during periods of stimulation, has yet to be determined. In this regard—and following published work [31,39,41,42,44,46]—ruthenium red was used in the experiment described in Figure 6 as an inhibitor of TRPV5/TRPV6 channels. However, ruthenium red is known to also inhibit intracellular release of calcium from sarcoplasmic reticulum as well as mitochondria [49]. Modulation of intracellular calcium release by ruthenium red should not affect results in this study because ruthenium red was added exclusively to the extracellular medium. Ruthenium red is also reported to block voltage-gated calcium channels [49]. At this time we cannot rule out the possibility that some of the effects seen in Figure 6 were due to inhibition of calcium influx through voltage-gated calcium channels.

RPE catalyzes a net, apical-to-basal, transepithelial calcium flux [6]. This flux could be mediated by paracellular transport and by transcellular flux crossing apical and basal membranes. The present work indicated that TRPV5 and TRPV6 channels are expressed on both apical and basal plasma membranes in native adult human RPE. Calcium influx through these channels could thus contribute to transcellular, unidirectional, apical-to-basal or basal-to-apical calcium flux. Channel insertion into either membrane would provide a mechanism to regulate the relative magnitude of net transepithelial flux.

The RPE plays a critical role in regulation of the SRS calcium concentration [3,5]. This is notable since, due to light dependent changes in photoreceptor calcium permeability, the SRS calcium content will change with light exposure. Apical plasma membrane-expressed TRPV5 or TRPV6 could contribute to regulation of the SRS calcium content. In this regard, the present work (Figure 6) and work in keratinocytes [47] show that TRPV channel activity can increase in response to elevation in extracellular calcium. Many different second-messenger systems in various cell types have been shown to regulate TRP channel activity [41-43,45]. In particular, the extracellular calcium-sensing receptor has been shown to modulate TRPV5 activity [48]. Further work in the RPE is required to determine which signals couple increased extracellular calcium concentration to increased TRPV channel activity.
